# The impact of three progressively introduced interventions on second wave daily COVID-19 case numbers in Melbourne, Australia

**DOI:** 10.1186/s12879-022-07502-3

**Published:** 2022-06-02

**Authors:** Allan Saul, Nick Scott, Tim Spelman, Brendan S. Crabb, Margaret Hellard

**Affiliations:** 1 The Burnet Institute, Melbourne, Australia; 2 Department of Epidemiology and Preventive Medicine, Monash University, Melbourne, Australia; 3 Department of Immunology and Pathology, Monash University, Melbourne, Australia; 4 Doherty Institute and School of Population and Global Health, University of Melbourne, Parkville, Australia; 5 Department of Infectious Diseases, The Alfred Hospital, Melbourne, Australia

**Keywords:** COVID-19, Regression analysis, Exponential, Prediction

## Abstract

**Background:**

The city of Melbourne, Australia experienced two waves of the COVID-19 epidemic peaking, the first in March and a more substantial wave in July 2020. During the second wave, a series of control measure were progressively introduced that initially slowed the growth of the epidemic then resulted in decreasing cases until there was no detectable local transmission.

**Methods:**

To determine the relative efficacy of the progressively introduced intervention measures, we modelled the second wave as a series of exponential growth and decay curves. We used a linear regression of the log of daily cases vs time, using a four-segment linear spline model corresponding to implementation of the three successive major public health measures. The primary model used all reported cases between 14 June and 15 September 2020 then compared the projection of the model with observed cases predicting future case trajectory up until the 31 October 2020 to assess the use of exponential models in projecting the future course and planning future interventions. The main outcome measures were the exponential daily growth constants, analysis of residuals and estimates of the 95% confidence intervals for the expected case distributions, comparison of predicted daily cases.

**Results:**

The exponential growth/decay constants in the primary analysis were: 0.122 (s.e. 0.004), 0.035 (s.e. 0.005), − 0.037 (s.e. 0.011), and − 0.069 (s.e. 0.003) for the initial growth rate, Stage 3, Stage 3 + compulsory masks and Stage 4, respectively. Extrapolation of the regression model from the 14 September to the 31 October matched the decline in observed cases over this period.

**Conclusions:**

The four-segment exponential model provided an excellent fit of the observed reported case data and predicted the day-to-day range of expected cases. The extrapolated regression accurately predicted the decline leading to epidemic control in Melbourne.

**Supplementary Information:**

The online version contains supplementary material available at 10.1186/s12879-022-07502-3.

## Background

Australia experienced a rise in coronavirus disease (COVID-19) cases in early 2020, peaking on 28 March 2020 and then declining in April after federal and state governments introduced strict community controls, travel bans and quarantining of international arrivals [1]. There was a resurgence of COVID-19 cases in the Australian state of Victoria starting in June, with almost all COVID-19 cases (95%) occurring in people living in the Victoria’s capital Melbourne and surrounding area, a population of 4.93 million out of the total Victorian population of 6.36 million. The resurgence led to the Victorian Government progressively introducing a series of control measures. These measures led to an initial slowing of the growth of the epidemic, then a rapid decline in cases and eventual local epidemic control (i.e., no observed cases for ≥ 28 days). New diagnoses of COVID-19 in Melbourne peaked at 652 cases on the 4 August 2020. The last diagnosed case associated with this resurgence was reported on the 28 October 2020. A description of this “second wave”, and associated control measure and outcomes has been published [1]. As determined from the sequences of 12,320 SARS-CoV-2 isolates (collected from 1 May 2020 to 30 September 2020) submitted to the GISAID database, 97.7% of circulating virus was the D.2 lineage of the GR clade [2]. This variant is also classified as B.1.1.25.5. It became the dominant variant in mid-June 2020 [3].

The restrictions were primarily applied to metropolitan Melbourne and the adjoining shire of Mitchell (hereafter “Melbourne”), although some were applied state-wide. In Melbourne, from June to October 2020 there were five main stages of the restrictions: (1) “pre-Stage 3” (2) “Stage 3”; (3) “Stage 3 + masks”; (4) “Stage 4”; and (5) a gradual easing of restrictions (Table 1). These major directives were accompanied by a vigorous testing of symptomatic people, extensive contact tracing. Infected people underwent 14-day isolation and similarly there were 14 days quarantining of close contacts, and this period was extended if the individuals did not return negative COVID-19 at the end of the isolation/quarantine period. As this policy was unchanged during the period covered by this study it is not specified in Table 1.

**Table 1:** Summary of COVID-19 restrictions in Melbourne during 2020

Date:	Directions
June 21 at 11:59 pm	“Pre-Stage 3” Relaxation of earlier restrictions enforced during first COVID-19 wave, retaining some restrictions including restrictions on visits to age care and health facilities and on the number of people in private gatherings (5), public gatherings (10) except for weddings (20) and funerals (50). It imposed a one person per 4 sq meter rule for pubs, bars, restaurants etc. [5]
July 2 at 11:59 pm	“Stage 3” restrictions to the 10 postcodes in Melbourne with the highest case burden (Stage 3 included: work or study from home where possible: closure of pubs, bars, entertainment venues, churches/places of worship; restricting restaurants and cafes to take-away only, and limiting public gatherings to two people) [6]
July 8 at 11:59 pm	Extension of Stage 3 restrictions introduced to the rest of Melbourne including the adjoining Mitchell Shire [7, 8]
July 20	Government announces that the mandatory use of masks or face coverings will be introduced in public settings for Melbourne on the July 22 at 11.59 pm [9]
July 22 at 11:59 pm	Addition of Mandatory use of masks or face coverings in public settings to Stage 3 restrictions for Melbourne [10]
August 2 at 6:00 pm	“Stage 4” restrictions for Melbourne (excluding Mitchell Shire). In addition to Stage 3 restrictions, they include: Curfew 8:00 pm to 5:00 am, restriction on movement to < 5 km, substantially increased restrictions on businesses and closure of non-essential stores, no visitor to homes [11, 12]
September 13th	Step 1 of eased restrictions. Very minor easing: Curfew changed from 8:00 to 9:00 pm. People living alone can have single visitor allowed in homes, playgrounds re-opened [13, 14]
October 18	Step 2 of eased restrictions. Minor easing: Curfew scrapped, limited opening of schools, some restrictions on non-essential businesses relaxed. Outdoor meetings with 10 people permitted [13]

This wave of COVID-19 took place prior to the registration and deployment of vaccines and prior to the designation of the Alpha Variant, the first “Variant of Concern”, by the World Health Organization on the 18 December 2020 [4].

A previous regression analysis showed that the introduction of Stage 3 restrictions was associated with a statistically significant reduction in the epidemic growth rate (reduction in the instantaneous growth rate from 0.14 per day to 0.034 per day, p < 10^–9^), but it was insufficient to stop the epidemic expansion [15]. Similarly, the introduction of mandatory masks in addition to Stage 3 restrictions, was associated with an exponential but slow decline in cases (instantaneous growth rate declined from 0.04 per day to − 0.023 per day, p = 0.006) [16]. Detailed information on the impact of the introduction of masks, including the level of uptake of mask usage and the impact of potentially confounding factors e.g., infections in health care workers, changed movement patterns, changing daily temperature, have been previously reported and therefore are not further extensively considered for the period covered by the former study [16]. The incremental impact of Stage 4 restrictions had not been previously assessed. These two prior analyses considered restricted time-periods over the epidemic wave (from 14 June to 7 July 2020, and from 10 July to 10 August 2020, respectively), with a common theme being that simple exponential models provided excellent fits to the time series of daily detected cases in the first three stages of restrictions (i.e., linear models fitted the logarithm of daily detected cases).

In this analysis, we undertook an interrupted time series analysis [17] of the combined time-series of detected cases across the four restriction phases (pre-Stage 3; Stage 3; Stage 3 + masks and Stage 4) to estimate and compare:

1. The incremental impact of each set of restrictions on the epidemic growth/decline rate. This expands previous analyses by estimation of the potential impact of Stage 4 restrictions and provides better estimates of the epidemic growth/decline in all stages due to the use of a larger data set;
2. The utility of the exponential regression model to describe the daily ranges in observed cases; and
3. The validity of using simple exponential model to predict future trends in daily cases as a tool for planning interventions.

The findings of this work are of considerable interest. At the time of this outbreak in 2020, Melbourne is one of the few cities globally that experienced a significant outbreak of COVID-19 and then regained control of the epidemic following the introduction of increasing government restrictions and mask use. Although vaccines and anti-viral therapies are now available, with the evolution of new variants (e.g., Omicron) that are not as well controlled by vaccination, the results of the controls introduced in 2020 are becoming relevant once more.

## Methods

### Data

Daily diagnosed cases for each Local Government Area (LGA) from 25 Jan 2020 to 20 Nov 2020 were downloaded on the 21 Nov 2020 as the NCOV_COVID_Cases_by_LGA_Source_20201120.cvs file from the Victorian Department of Health and Human Services (DHHS) website [18]. Infections in people living outside of metropolitan Melbourne or acquired overseas were excluded.

Daily maximum and minimum temperatures for Melbourne Olympic Park, (Latitude: 37.83° S · Longitude: 144.98° E Elevation: 8 m) for 2020 were obtained from the Australian Bureau of Meteorology historical data site [19].

### Time-period of interest

The model was based on reported cases from 14 June to 14 September 2020 inclusive, and hence covers epidemic growth/decline in four distinct phases of restrictions: “pre-Stage 3”, “Stage 3”, “Stage 3 + masks”, and “Stage 4”.

**Fig. 1:**
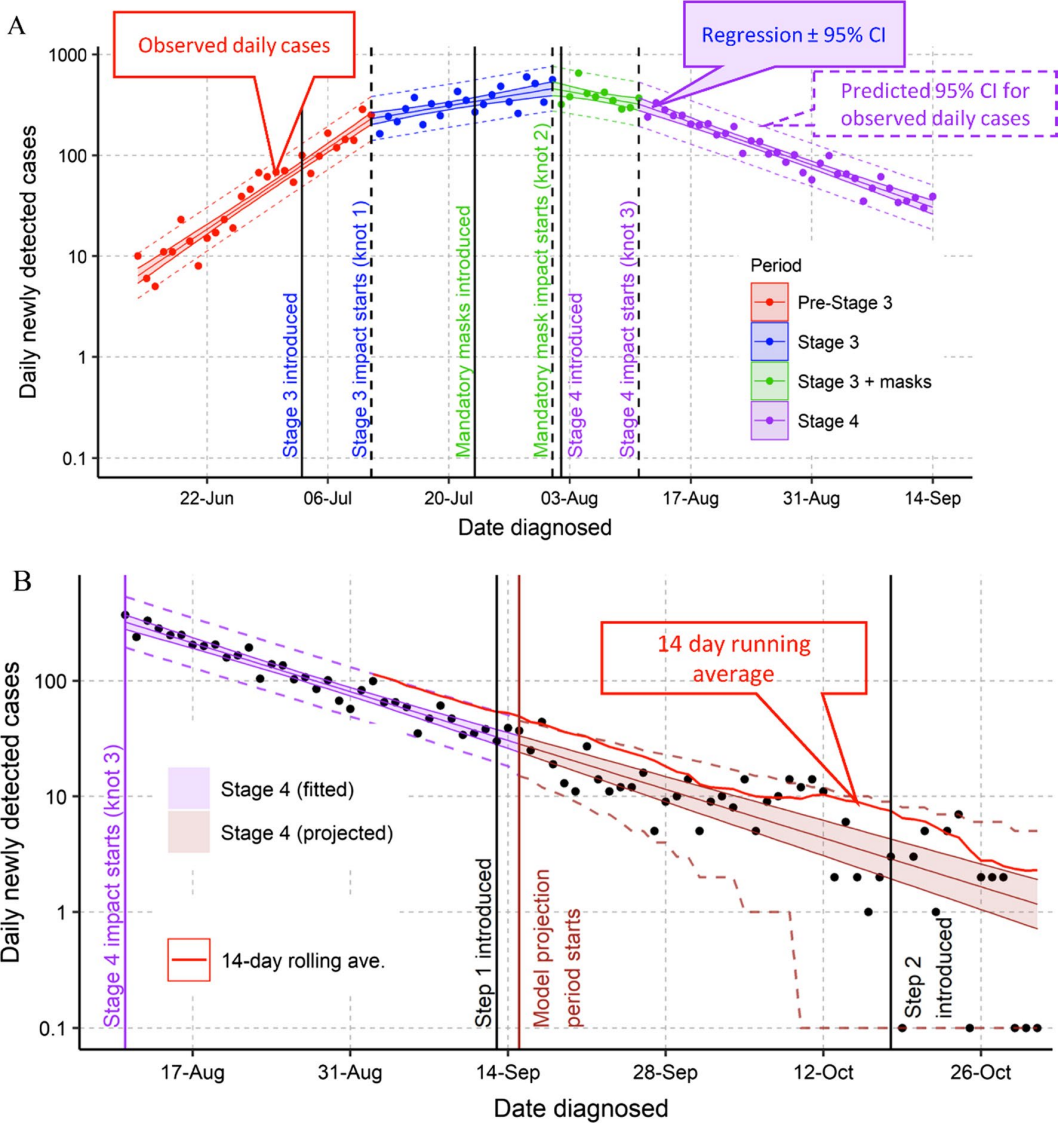
Regression analysis of ln(daily cases) as a function of date of diagnosis for **A** the period analysed (14 June to 14 September 2020) and **B** extrapolated from the 15 September to 31 October 2020

The 14 June starting day was selected to match the previous study on the introduction of “Stage 3” restrictions in Melbourne [15] and because at earlier dates, the daily case numbers were so low, resulting in a high variance during this period that could bias the model (specifically there was significant heteroskedasticity at earlier times, see Additional file 1). On the 14 September 2020, a decision was made to stop the periodic updates of the model. By this date, there was a clear downward trend in daily cases and stopping the analysis on the 14 September allowed testing the ability of the regression model to predict future cases. The primary analysis reported in this paper (Fig. 1A) was conducted on the 14 September 2020.

### Statistical model

Based on previous observations that the growth of the COVID-19 epidemic fitted an exponential model, with the introduction of public health interventions resulting in a change in growth rate following a defined delay [15, 16], an interrupted time series model was chosen prior to the analysis. Specifically this assumed slope change following a defined lag is the model described in Bernal et al., Fig. 2D [17]. This was both consistent with the earlier observations, but also the simplest model that could fit, requiring estimation of 5 parameters: a starting daily case rate and a single exponential growth parameter for the pre-intervention period and one following each change in restrictions.

The growth or decay rates were estimated using a non-weighted linear regression to fit the natural logarithm of the daily cases against time, using a linear spline model with three knots with the lspline R routine [20]. The three knots divide the model into 4 linked linear segments with the knot days (i.e., the day on which the linear segments are “tied” together) corresponding to the times at which impact of successively introduced control procedures are predicted to start. The knots allow deflections in the line of best fit due to the impact of the introduction of different restrictions. The assumption of linearity of the four segments, and hence the presumption of an exponential growth/decay was tested by assessing whether studentized residuals were normally distributed and independent of the time (i.e., no significant heteroskedasticity). Due to the generation interval for the SARS-CoV-2 variants circulating at the time (a mean of 4–6 days [21, 22]) and delays in testing and reporting, there is an expected delay between the introduction of restrictions and observing them in diagnosis data. The knot days were initially estimated to be 8 days following the introduction of new control measures; for example, the introduction of Stage 3 restrictions took place at midnight on the 2 July, so the knot day was 3 July + 8 days = 11 July. This delay was based on a previous study that estimated an 8-day delay between the introduction of mandatory face masks in Melbourne and the change in the epidemic growth rate [16]. As in that previous study, sensitivity analyses were conducted following the fit to maximize the model fit (as quantified via the adjusted R^2^) by varying the delay between the introduction of new restrictions and the knot day.

This analysis estimated the instantaneous growth constant (*k*) (i.e., the slope of the ln(daily cases) vs time regression) for each segment and the difference between these growth constants was used as the primary measure to test for a significant association between successive public health measures being introduced and subsequent changes in growth/decay rates.

The equation for the regression is

$${y}_{i}={\epsilon }_{i}+{\beta }_{0}+{\beta }_{1}{x}_{i}+\sum_{k=1}^{3}{b}_{k}{\left({x}_{i}-{\alpha }_{k}\right)}_{+},$$

where $$\left( {x_{i} - \alpha_{k} } \right)_{ + } = \left\{ {\begin{array}{*{20}c} {0,} & {x_{i} < \alpha_{k} } \\ {\left( {x_{i} - \alpha_{k} } \right),} & {x_{i} \ge \alpha_{k} } \\ \end{array} } \right.$$ and *β*_0_, *β*_1_, *b*_1_, *b*_2_, *b*_3_ are the regression coefficients; *α*_1_, *α*_2_, and *α*_3_ are the knot days relative to the data start date; *y*_*i*_ were the log of diagnoses observed on day *x*_i_ and *ε*_i_ is an error term.

The doubling/halving time of the epidemic was calculated as *t*_2_ = ln(2)/*k.*

A secondary analysis was performed on the residuals from the three knot, lspline model to test whether any significant inflection occurred that was not consistent with the introduction of the successive restrictions on the 2nd July, 22nd July and 2nd August 2020 using a Joinpoint method [23] as implemented by the Joinpoint routine version 4.9.0.1 [24].

### Effective reproduction ratio (R_eff_)

We calculated *R*_*eff*_ as the ratio of new cases separated by one serial interval for SARS-CoV-2 infections using the Lotka–Euler equation [25]. Due to uncertainties in the serial interval, we estimate a range for *R*_*eff*_ based on two measurements of the serial interval: a normal distribution with mean 3.96, standard deviation 4.75 days based on 468 pairs of infections in China [21], and a gamma distribution with mean 6.49, standard deviation 4.90 days based on 1015 pairs of infections in China, Japan and Singapore [22]. We include changes in estimated *R*_*eff*_ as a secondary estimate to allow comparisons with other studies but emphasize that the estimates of *R*_*eff*_ are subject to uncertainty in estimates of the generation interval (approximated as the serial interval) and its distribution.

We also use the change in *R*_*eff*_ as a measure of the relative impact of successively increasing control measures for both the 3.96 and the 6.49-day serial interval estimates.

### Extension to 31 October 2020

To test the utility of the regression analysis to predict future trends, we extrapolated the regression line from the last day of data fitted (14 September 2020) to the end of October 2020. We calculated the predicted 95% range of daily cases in this period assuming an inverse Poisson distribution using two methods: the estimated daily cases calculated as the antilog of the mean ln(cases) or the mean ln(cases) ± the 95% confidence intervals from the regression line, to provide minimum and maximum estimates of the range, respectively.

## Results

### Regression analysis 14 July to 14 September 2020

The regression analysis of the ln(daily cases) vs time gave a fit consistent with a 3-knot model. Sensitivity analysis (Additional file 1: Table S1) gave the best fit for an 8-day delay for the first and third knot. For the knot following introduction of masks, the fit was similar for a 9 or 10 day delay (adj R^2^ for 8, 9, 10 and 11 day delays were 0.9565, 0.9584, 0.9584 and 0.9580, respectively) and the 9 day fit was chosen [16]. This is a 1 day longer delay than the 8-day delay found from the previous study that considered only the transition accompanying mask introduction. The resulting model is shown in Fig. 1A for the model with four log-linear segments joined by three knots with each knot 8 or 9 days after the introduction of the increased control measures (Fig. 1).

For each log-linear segment, the corresponding instantaneous growth rate, doubling/having time and *R*_*eff*_ are shown are shown in Table 2, alongside the percentage reduction in *R*_*eff*_ between segments. These growth constants translate into from a 14% daily increase in pre-stage 3 to a 3.3% increase in daily cases during of Stage 3, then to a 3.5% decrease in daily cases after introduction of mandatory masks and a further a 6.7% decrease in Stage 4.

**Table 2:** Growth constants, doubling times and R_eff_ for each stage

Control level	Growth constant per day	p > 0	p *k*_*n*_ − *k*_*n*−1_ > 0	Doubling/halving time (95% CI) days	Serial interval 3.96		Serial interval 6.49	
					*R*_*eff*_ (95% C.I.)	Successive reduction	*R*_*eff*_ (95% C.I.)	Successive reduction
Pre-Stage 3	0.133 (s.e. 0.005)	< 0.001	–	5.22 (4.87 to 5.61)	1.39 (1.37 to 1.40)	–	2.02 (1.93 to 2.1)	–
Stage 3	0.033 (s.e. 0.006)	< 0.001	< 0.001	21.21 (15.75 to 32.49)	1.12 (1.08 to 1.16)	18.5%	1.22 (1.14 to 1.3)	35.4%
Stage 3 + masks	− 0.036 (s.e. 0.012)	0.003	< 0.001	− 19.47 (− 54.63 to − 11.84)	0.86 (0.76 to 0.95)	25.9%	0.78 (0.65 to 0.92)	39.0%
Stage 4	− 0.069 (s.e. 0.004)	< 0.001	0.019	− 10 (− 11.19 to − 9.03)	0.72 (0.69 to 0.75)	14.7%	0.59 (0.56 to 0.63)	22.6%

Intercept: 1.86 s.e. 0.086; Residual standard error: 0.24 on 88 degrees of freedom; Multiple R-squared: 0.959 Adjusted R-squared: 0.957; F-statistic: 509 on 4 and 88 DF, p-value: < 0.001. Doubling/halving time calculated as ln(2)/k (see text)

Over the whole of the regression, the observed daily cases were only outside the 95% predicted confidence limits for three of the 93 days analysed. I.e., the distribution fitted on 96% of the days as expected for a 95% confidence interval.

The 4-segment linear exponential model gave a good fit of the observed data to the model as judged by the goodness-of fit tests. These tests as well as the statistical tests of the relevant null hypotheses are detailed in Additional file 1, but to summarize: the distribution of studentized residual values of the log transformed cases was indistinguishable from a normal distribution (QQ plot and Shapiro–Wilk Test, p = 0.73). There was no detectible autocorrelation (Durbin–Watson Test) and no outlier data that significantly impacted on the regression (maximum Cook’s D value was 0.085). However, one test showed a deviation from an ideal regression: there was evidence of heteroskedasticity (Breusch–Pagan test p = 0.0013, White test p = 0.0014). Inspection of the residuals indicted that the initial data points had a higher scatter consistent with the lower daily cases in June. A regression using three segments with two knots for the period 11 July to 14 September (i.e., starting at the day of the first expected impact of Stage 3) gave a fit with no marked heteroskedasticity (i.e. p = 0.11) and the growth constants for the stage 3, stage 3 + masks and stage 4 were essentially unchanged from those obtained with the full model (Table 2). Thus, the observed heteroskedasticity at the start of the first segment had no likely impact on the estimates of growth constants for Stage 3, Stage 3 + masks or Stage 4.

Consistent with these goodness-of-fit tests, no additional inflection point was found by analysing the residuals from the lspline fit regression using Joinpoint and the slope of the unjoined line fitted by Joinpoint was 0.00. i.e., there was no unaccounted residual slope.

### Extrapolation to the 31 October 2020

The observed distribution of cases over the period 15 September to 31 October closely matched the projected distribution, with most cases lying within the 95% confidence intervals. Importantly, this projection showed a high probability of zero cases at the end of October (31% probability of zero cases on the 31 October). Since the confidence interval for the regression line was small compared to the Poisson confidence intervals, there was little difference in the confidence interval calculated with the two methods based on the mean or the mean ± 95% confidence interval.

### Temperature changes during the study period

Minima and maxima temperature for a site in the centre of Melbourne [19], show substantial scatter, but the underlying temperature trend shows little variation over the study time (Additional file 1: Fig S4) with the biggest temperature difference of 1.9 °C and 2.4 °C for minimum and maximum temperatures, respectively, from mid-July to the last day in the regression analysis (14th September 2020).

## Discussion

From 14 June to 14 September 2020, the four-segment linear model gave an excellent fit (adjusted R^2^ = 0.958) to the observed daily cases in Melbourne. There was a highly significant change in the growth constants (and associated *R*_*eff*_) following the three major changes in control measures. The biggest successive impact as measured by the successive percent change in *R*_*eff*_, was the addition of masks to the existing Stage 3 restrictions, followed by increased restrictions at the start Stage 3, regardless of the underlying assumptions about serial interval (Table 2).

Although introduction of masks had the largest effect, the addition of further restrictions at the start of Stage 4 still was associated with a statistically significant change in the growth constant. Without Stage 4, the extrapolated trend predicted 18 new cases per day on the 31 October compared to the 1.2 cases predicted with the additional Stage 4 restrictions. Alternatively, it would have taken until the 15 January to reach an average of 1.2 cases per day. Also, the addition of Stage 4 is estimated to have prevented approximately 3900 cases compared to continued use of just Stage 3 + masks until local epidemic control.

Growth constants and associated *R*_*eff*_ had previously been reported using regression analyses for the initial growth period and Stage 3 for all cases in Victoria [15, 16] and for Stage 3 with and without masks for Melbourne cases [16]. The analyses in this paper used a wider range of data and importantly, included the additional Stage 4 restrictions: The first study fitted the data to two independent linear regression lines of the ln(daily cases) vs time and the second to a model with two linear segments and a single knot. As a result of the larger database and by constraining the end of each segment through the use of the knots, the precision of the estimates are improved as judged by the smaller standard errors of the estimated growth constants in the current study compared to the earlier studies (Table 3).

**Table 3:** Comparison of coefficients from previous studies with the current study

	Pre-Stage 3			Stage 3			Stage 3 + Masks		
	Growth constant	Std. error	*R*_*eff*_	Growth constant	Std. error	*R*_*eff*_	Growth constant	Std. error	*R*_*eff*_
Victoria [15]	0.14	0.008	1.74	0.038	0.009	1.16			
Melbourne [16]				0.042	0.007	1.18	− 0.023	0.017	0.91
Melbourne, this study	0.133	0.005	1.39	0.033	0.006	1.12	− 0.036	0.012	0.86

*R*_*eff*_ Calculated assuming a 3.96 day serial interval

This study extends the information on the delay from our earlier studies [15, 16] between introducing an additional control and an observed change in the growth or decline in the daily case numbers. Sensitivity analyses for the impact on the overall fit as judged by changes in the adjusted R^2^ when the assumed delay is changed are shown in Additional file 1: Table S1. We assume that the best fit in the model delay for the period between introduction of Stage 3 and Stage 4 corresponds to the maximum adjusted R^2^, although no formal statistical test has been done to determine the statistical significance of changes in this parameter. With this assumption the best fit for a change in the observed growth rate was 8 days. For the introduction of masks, the best fit was at 9 days compared to the earlier estimate of 8 days [16], with differences in best fit likely due to the availability of additional data to further constrain the model. These delays are consistent with the expected delays associated with the generation time of the virus and delays in testing and reporting new cases.

The interrupted time series analysis [17] used in this and the previous study [16] is particularly suited for looking at impact of public health interventions during an epidemic. In this study we have primarily used the lspline with knot analysis [20] supplemented with the Joinpoint routine [23] to look for residual changes not addressed in the lspline model. Both use multiple linear regression of log transformed daily cases to test exponential growth and decay, but differ by an important characteristic: the lspline algorithm tests the hypothesis that there is a significant change in the exponential growth constant associated with introduction of a specific control measure. The Joinpoint is descriptive and tests if a change in growth rates have occurred at any non-specified time.

The interrupted time series with lspline analysis can be implemented rapidly and does not have the ethics issues associated with withholding a potential effective measure during an epidemic; for example, withholding masks from whole communities as required for a block randomized trial. The analysis uses the population prior to introduction of new restrictions as the control for the post-restriction outcome. This has both strengths and weaknesses. Because it uses the whole population, it is less sensitive to sampling biases and with a population in excess of 5 million, it has a high power of detecting an effect. On the other hand, there are two important potential weaknesses. First, the ability to measure the change in growth rates for a series of interventions depends on how quickly each successive intervention is introduced in the whole community. A simultaneously introduction across the whole community should give rise to a sharp transition in the growth rates as was seen in this study. Second, the analysis is subject to biases caused by events occurring contemporaneously with the introduced interventions that may impact the estimated size of the change, or in extreme cases, lead to no significant or false positive associations.

Introduction of the three interventions assessed in this study (Stage 3, masks and Stage 4) was accompanied by substantial publicity, and strong policing with initial warnings, then substantial fines for people not complying and there is no reason to believe that the interventions were not implemented rapidly across the whole population. In the case of mask introduction, as shown in the previous publication, a major and rapid change in compliance with mask usage was explicitly observed [16].

The Stage 3 controls were introduced in two stages, 6 days apart (at midnight on the 2nd and 8th June 2020) with the first stage covering the areas within Melbourne containing almost all cases. An lspline analysis with an extra knot on the 17th June (8 days after the second phase of the Stage 3 introduction) failed to find any significant change associated with this second introduction and Joinpoint analysis of the residuals of the model with 3 knot model also failed to find any further deflection points suggesting the extension to the wider metropolitan area had no significant impact at that time. Thus, the available information on implementation and this secondary analysis suggests that there was a rapid and simultaneous introduction of the three interventions across Greater Melbourne.

Confounding factors that occurred rapidly, nearly simultaneously over the study area and that occurred at a similar time to introduction of controls could impact this analysis. There was an extensive analysis of possible confounders in the previous analysis of the impact the introduction of masks [16]. Briefly, that study considered the impact of COVID-19 cases on testing patterns, infections in health care workers, the impact of changed (reduced) movement patterns and changing daily temperature. None of the potential confounding factors had a significant impact on the estimated changes in the growth constant going from Stage 3 to Stage 3 + Masks and were not considered in this broader study. As concluded in the previous work, although no statistically significant impact was observed, these confounding factors may result in an under-estimate of the impact of each of the increased restrictions. For example, health care workers were already using masks and other PPE over the whole period.

The current study covers a longer period than the previous analysis—nearly 5 months from the Southern hemisphere’s early winter to late spring. Several publications have suggested that seasonal changes or different climates may impact on *R*_*eff*_ [26, 27]. For example, studies undertaken during 2020 suggest a significant correlation of the estimated *R*_*0*_ of the first strains circulating in different countries correlate with latitude and the implication that this was a climate effect. One of these studies (Frontal et al. [27]) correlated daily mean temperature at different locations with *R*_*0*_ and showed evidence for changing *R*_*eff*_ within a single location with changing temperature that impacted on the timing of new waves of infection. The size of the temperature effect can be estimated from the data in Frontal et al., extended Fig. 1B. The effect is small: a change in *R*_*0*_ of 0.017 per °C and would result in an undetectable change of only 0.04 in the estimated *R*_*eff*_. There was a larger temperature (4 °C) increase from 14 Sept 2020 to the end of the study on the 31 October 2020 for a predicted decrease in *R*_*eff*_ of 0.07. The data for these days were not part of the regression, and the temperature increase was greater than at other times, so temperature effects may be easier to detect in this projection. Even in this period, with the predicted change in *R*_*eff*_ of 0.07, there was no evidence of a temperature effect.

There are multiple other factors that may impact transmission of SARS-CoV-2. For example, humidity, precipitation, wind speed, ultraviolet index, air pollution, seasonal comorbidities [28]. However, to have impacted this study, one or more of these factors would both need to have a substantial effect on transmission and also have a major change in level, intensity etc., exactly co-incident with the introduction of the Stage 3, the mask mandate or Stage 4. These have not been formally tested are unlikely to have been important. While it is not possible to rule out these or other unknown confounding factors, this analysis is consistent with the hypotheses that there was a highly statistically significant change in the exponential growth rates of the COVID-19 epidemic in Melbourne associated with the introduction of the Stage 3, Masks and Stage 4 controls.

Our results have several implications for real time control decisions to guide the future imposition or relaxation of control measures. These include:

1. Managing community expectation in the fluctuations of observed daily cases. The power of the statistics associated with a regression analysis provides an estimate of the expected daily range. As shown in Fig. 1, this analysis accurately predicted the range of daily cases expected (i.e., for the main analysis period, for 90 out of 93 days (96%), the observed distribution lay within the 95% estimated range). It will give a short-term estimate of expected range. If applied in future outbreaks may give either reassurance or a rapid warning that the epidemic situation is changing.
2. Longer term prediction of future trends. Extrapolation of the regression analysis from the 14 September to the 31 October 2020 correctly predicted the trend of the epidemic. This approach differs from the 14-day rolling average approach used in Victoria to provide Go/No Go decision on relaxing controls [1]. Because the regression analysis is based on all available data and built around a statistical model, it can be projected into the future with realistic confidence ranges. The rolling average is based on much less data (14 days) and is subject to greater variation (Fig. 1B) and gives an historical view on where the epidemic was on average 7-days previously. It cannot provide estimates of the expected daily fluctuations in case number or a statistical basis including confidence ranges on predicting future cases.
3. Quantitative data on the impact of progressively imposed control measures. As judged by *R*_*eff*_, this study highlights that the single most important sequential control measure was consistent with the addition of mandatory masks. The much more stringent control measures subsequently imposed with Stage 4 had a significant but smaller impact on the time to epidemic control but at a much greater cost to the community. If future, with a much smaller outbreak, Stage 3 + masks alone may be sufficient. Alternatively, if faced with a more highly transmissible strain [29] the data from this study may be useful in calibrating future models of what will be required for local epidemic control.
4. As expected from the fit between daily cases and the model (Fig. 1), the formal goodness of fit tests (Additional file 1) and a Joinpoint analysis of the residuals, there was no other unexplained change in the growth constants.

Extrapolation of these results from Melbourne, Australia to other COVID infected locations needs to be done with caution. For example, taking into account different climatic [26, 27] and socio-economic factors [30, 31].

### Postscript

The D2 peak in described in this paper in Melbourne during 2020, had a peak 7-day running average of 590 cases diagnosed per day on the 2 August 2020 and a 7-day average peak of 16 deaths per day on the 14 August 2020. Following the period analysed in this paper, there were a series of further COVID outbreaks in the eastern states of Australia.

In 2021, there was a major Delta VOC outbreak in New South Wales, the Australian Capital Territory and Victoria peaking with an Australian 7-day running average of 2280 daily cases diagnosed on the 15 October 2021 and an Australian 7-day running average of 15.3 deaths per day on the 15 October [32, 33].

By the 31 December 2021, Australia had achieved high vaccination rates with 91% of the population 16 years and older double vaccinated and 75% of the 12- to 15-year-old children also double vaccinated [34]. At this time, Australian States and Territories except Western Australia, removed almost all public health restrictions, relying on vaccination to prevent serious disease and death.

Subsequently there was a large outbreak of Omicron BA.1 with an Australian peak 7-day running average of 108,900 diagnosed cases per day on the 11 January 2022 and a 7-day running average peak of 88 deaths per day on the 27 January 2022 [32, 33]. Daily cases subsequently dropped reaching an Australian nadir on the 6 February 2022. Since that time, case number have steadily risen again, predominantly with Omicron BA.2 infection [3]. At the time of writing (1 April 2022) there is a 7-day average of 56,000 cases and 24 deaths per day. During March, there has been a marked weekly variation with diagnosed cases peaking on Thursday and a minimum on Sundays, and an underlying exponential increase with a daily growth constant of 0.032 per day. (Additional file 1: Fig S5).

There is a perception that vaccination has replaced the need for public health measures, but in the first 3 months of 2022, Australia has had 8 times more diagnosed cases of COVID (and thus more school/work absenteeism) than the whole of 2020 plus 2021 (4,115,000 vs 505,000 cases) and far more deaths (3741 vs 2253 deaths) [33]. Australia is now facing a second Omicron peak, predominantly caused by the BA.2 variant. Although it is claimed that this BA.2 variant is highly infectious, in the current epidemiological conditions with high levels of vaccination but minimal public heath controls, the daily growth rate of the outbreak at 0.032 per day (Additional file 1: Fig S5), is much smaller than the growth rate of 0.133 in the D2 outbreak in Melbourne in August 2020 Melbourne “pre-stage 3” period (Table 2) during which time there were more restrictions (stringent tracing and quarantine and density limits) than are currently imposed in Australia. The data presented in this paper, suggest that re-imposition of public health measures, even if limited, on top of existing vaccination would control this current outbreak with a major reduction in school and business absenteeism, hospital load and deaths.

## Conclusions

A simple and easily calculated linear regression model with knots at each significant change in control measures, gave an excellent fit to the observed daily cases in Melbourne’s second COVID-19 wave, providing quantitative data on the relatively impact of the different controls and provides a framework for simple models to guide future outbreaks responses..

## Supplementary Information

**Additional file 1.** Goodness of fit tests for the regression analysis, sensitivity analyses, temperature changes and Australian March 2022 COVID data.

## Data Availability

The dataset analysed in this study is publicly available from https://www.dhhs.vic.gov.au/ncov-covid-cases-by-lga-source-csv.

## Abbreviations

DHHS: Victorian Department of Health and Human Services
*k*: Instantaneous growth constant
*R*_0_: Basic reproduction ratio the theoretical reproduction ratio in a naïve population with no controls
*R*_*eff*_: Effective reproduction ratio in a population in the presence of control
s.e.: Standard error
C.I.: Confidence interval
