# Supplementary material for: The impact of three progressively introduced interventions on second wave daily COVID-19 case numbers in Melbourne, Australia
Source: BMC Infect Dis. 2022 Jun 2;22:514. doi: 10.1186/s12879-022-07502-3 (PMC9160504; doi:10.1186/s12879-022-07502-3)
Supplement: Supplementary file 1 — Additional file 1. Goodness of fit tests for the regression analysis, sensitivity analyses, temperature changes and Australian March 2022 COVID data. [file 12879_2022_7502_MOESM1_ESM.docx]

1. **Goodness of fit tests for regression analysis**

This supplement describes the tests undertaken on the regression analysis shown in Figure 1A of the main paper

In a valid multiple linear regression, it is expected that the residuals meet the following criteria

- **Linearity**: The*ε_i_*have mean of 0
- **Independence**: The*ε_i_* are independent
- **Normality**: The *ε_i_* are normally distributed
- **Homogeneity of variances**: The*ε_i_*have the same variance *σ*^2^

We primarily test using the Studentized residuals *ε_i_* since raw residuals are not expected to be completely independent but provide some test results with the raw residuals

**Linearity**

|  | Mean |
| --- | --- |
| Raw Residuals | 4.0 x 10^-16^ |

Expect zero. Pass

**Independence**

*Durban-Watson Test*

| Test Statistic | 2.040 |
| --- | --- |
| Critical lower limit for α = 0.05 | 1.574 |
| Critical upper limit for α = 0.05 | 1.753 |
|  |  |

Test value is greater than the critical upper limit so no positive or negative autocorrelation is detected. Therefore there is no evidence for lack of independence.

*Graphical display*

**
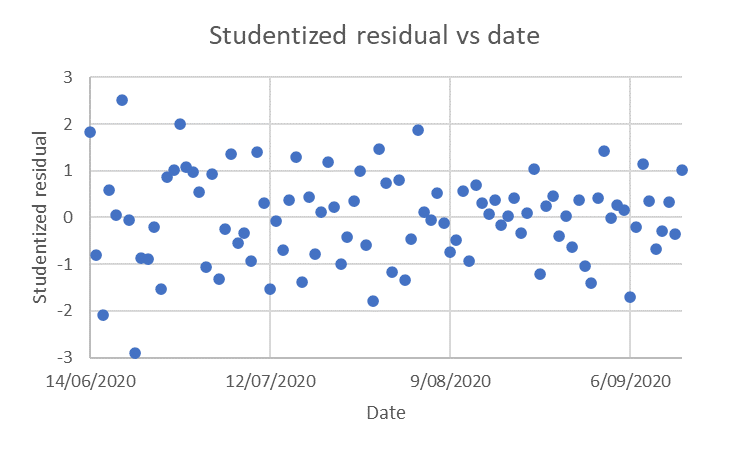
**

Fig. S1. Plot of Studentized residuals *versus* day of the study. The knot days were 11 July, 1 August and 12 August

**Normality**

*Shapiro-Wilk test*

|  | residual |  |
| --- | --- | --- |
| W-stat | 0.997 |  |
| p-value | 0.8321 |  |

Test passes for residuals (p>0.05)

*Graphical QQ Plot*


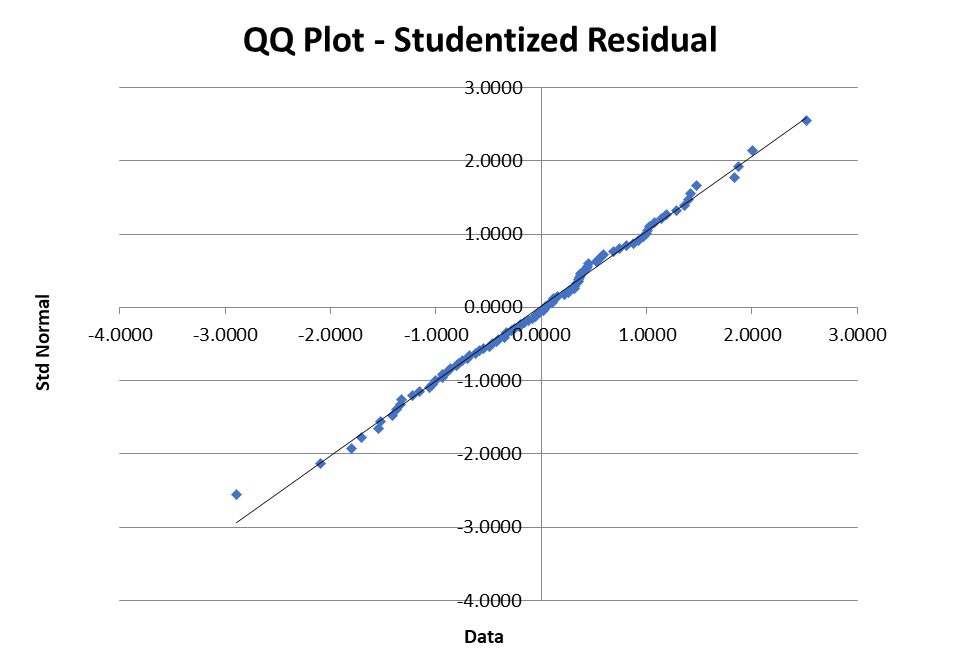


Fig. S2. QQ (Quantile-Quantile) plot of the ordered Studentized residuals vs a the corresponding quantiles of a normal distribution. Black line indicates the position of a perfectly normally distributed Studentized residuals.

**Homogeneity of Variances**

*Test for Heteroskedasticity with Breusch-Pagan test over the range 14 June to 14 September (i.e pre-Stage 3 to the end of the analysis in Stage 4)*

| Number of Days | 93 |
| --- | --- |
| No. of Independent variables | 4 |
| LM statistic | 17.81 |
| Degrees of Freedom | 2 |
| p-value | 0.0013 |

Pass criteria: p value >0.05. Fail

*Test for Heteroskedasticity with Breusch-Pagan test over the range 11 July to 14 September (i.e just Stage 3 to the end of the analysis in Stage 4)*

| Number of Days | 66 |
| --- | --- |
| No. of Independent variables | 4 |
| LM statistic | 7.57 |
| Degrees of Freedom | 2 |
| p-value | 0.11 |

Pass criteria: p value >0.05. pass

Also see Fig S1

**Other test**

Regression was tested for the infludence of outliers using Cook’s D test. As shown below, the maximum Cook’s D value was 0.109 on 16 June 2020 As this is much less than 1, there are no outliers with a significant impact on the regression


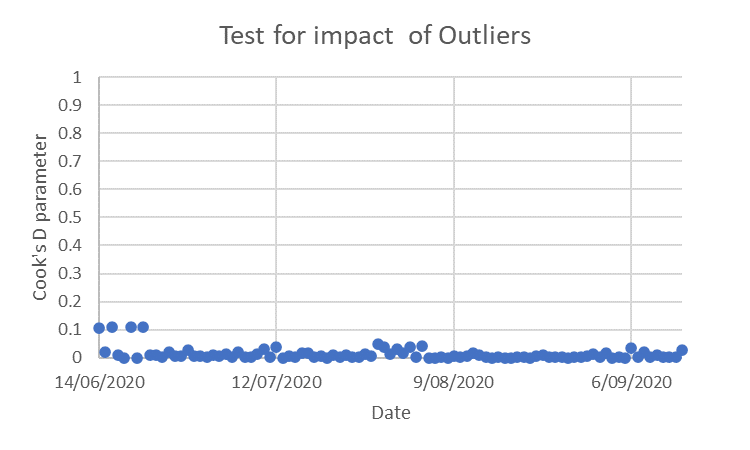


Fig. S3. Cook’s D parameter vs study day

**Table S1. Sensitivity analyses for delay**

| New control | Delay | Adjusted R^2^ |
| --- | --- | --- |
| Stage 3 (knot 1) | 6 | 0.9581 |
|  | 7 | 0.9570 |
|  | **8** | **0.9584** |
|  | 9 | 0.9558 |
|  | 10 | 0.9566 |
| Masks (knot 2) | 6 | 0.9561 |
|  | 7 | 0.9564 |
|  | 8 | 0.9565 |
|  | **9** | **0.9584** |
|  | 10 | 0.9584 |
|  | 11 | 0.9580 |
| Stage 4 (knot 3) | 6 | 0.9581 |
|  | 7 | 0.9581 |
|  | **8** | **0.9584** |
|  | 9 | 0.9583 |
|  | 10 | 0.9584 |
|  | 11 | 0.9566 |

**Temperature changes**

Daily maximum and minumum temperatures weredowloaded from the Australian Bureau of Meterology database for Melbourne Airport for calandar 2020. The underlying temperature trend was estimated by fitting sine curves to the 366 days of data to maximum and minimum temperatures. The daily mininum, maximum and fitted since curves are shown in Fig. S4. For the period from the start to the study (14 June 2020) to the end of the extension period (31 Oct 2020). There was less than a 0.2°C and a 0.3°C temperature change in the week before and after each of the knots for the trends in the minimum and maximum temperatures, respectively. There was a 4.3°C and 6.6°C increase in temperature in the underlying minimum and maximum temperatures, respectively between the first week of the “Stage 4” restrictions (starting 11 Aug 2020) and the last week of the observation (ending 31 Oct 2020).

Fig. S4. Daily temperatures for Melbourne Olympic Park for the period covering the regression analysis and the extension (14 June 2020 to 31^st^ October 2020). Orange dots: daily maximum; blue dots: daily minimim; orange line: fitted sine curve for daily maxima from 1 Jan 2020 to 31 Dec 2020; blue line: fitted sine curve for daily minima from 1 Jan 2020 to 31 Dec 2020. The lspline regression analysis covered the period 14 June 2020 (start of graph) to the 14 Sept 2020 (solid vertical black line).

**Postscript data**


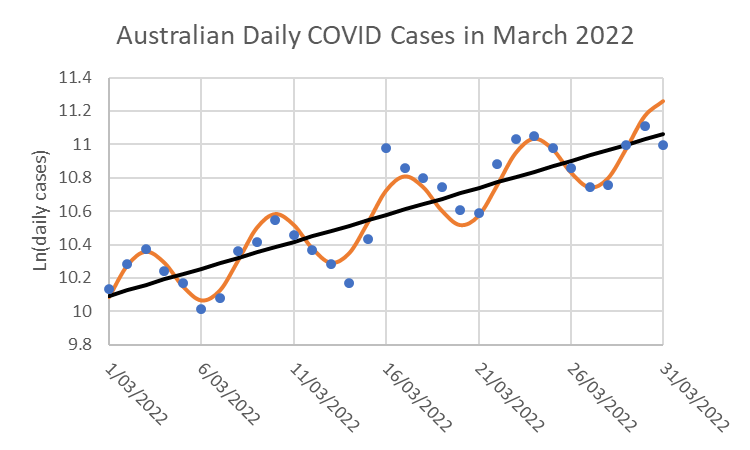


Fig. S5. Time course of Omicron March 2022 outbreak.

- Blue dots are the natural log of the total Australian daily cases vs date.
- Orange line. Best fit model of a sinusoidal weekly variation overlying an exponential increase: Ln(daily cases) = 10.06 + 0.032**d* - 0.201*sin(0.896**d* -4.09)
- Black line: underlying exponential increase: ln(daily cases) = 10.06 + 0.0323**d*

where *d* =1 is the 1st March 2022.

1. **Reference**

All tests were performed using the Excel routines available from

Zaiontz, C. 2020 Real Statistics Using Excel. Version 7.3.3 <https://www.real-statistics.com/>
